# Molecular detection of Aspergilli from commercial chicken in selected areas of Bangladesh

**DOI:** 10.5455/javar.2022.i583

**Published:** 2022-05-27

**Authors:** Md. Yeasin Arafat, Md. Monowarul Islam, Shamim Ahamed, Md. Muket Mahmud, Md. Bahanur Rahman, K.H.M. Nazmul Hussain Nazir

**Affiliations:** Department of Microbiology and Hygiene, Faculty of Veterinary Science, Bangladesh Agricultural University, Mymensingh, Bangladesh

**Keywords:** Aspergilli, A. flavus, A. fumigatus, A. niger, chicken, prevalence, PCR

## Abstract

**Objectives:**

This study was designed to isolate, identify, and determine the prevalence of *Aspergilli* in commercial chicken in selected areas of Bangladesh.

**Materials and Methods:**

A total of 50 lung samples from suspected dead chickens, comprising broilers (*n* = 32) and layers (*n* = 18), aged between 5 days and 45 weeks, were collected from poultry farms located in the Gazipur district in Bangladesh. Fungi were primarily identified based on the colony morphology using potato dextrose agar (PDA). DNA was extracted from the suspected colonies. *Aspegillus *spp. was detected by genus-specific ASAP-1 and ASAP-2. *Aspergillus *spp. were then screened by polymerase chain reaction targeting *Aspergillus flavus *(FLA-1 and FLA-2), *Aspergillus fumigatus *(ASPU and Af3r), and *Aspergillus niger *(ASPU and Nilr).

**Results:**

The overall prevalence of *Aspergillus *spp. was 44% (*n* = 22/50; *p < *0.05). Among the *Aspergilli*, *A. flavus *was detected in 10% (*n* = 5/50) of the samples. Similarly, *A. fumigatus* and *A. niger *were detected at 26% (*n* = 13/50) and 8% (*n* = 4/50) respectively. Three samples were associated with more than one fungus; two fungi (*A. flavus* and *A*. *niger*) were in two samples, and three fungi (*A. flavus*,* A. fumigatus*, and* A. niger*) were in one sample.

**Conclusion:**

Isolation and prevalence of *Aspergillus *spp. in commercial chicken were studied for the first time in Bangladesh.

## Introduction

Aspergillosis in the form of brooder pneumonia is a major concern that affects chickens, causing high economic losses due to its high morbidity and mortality in Bangladesh [1]. Avian aspergillosis is an infectious fungal disease characterized mainly by respiratory symptoms. This disease has been reported worldwide in a large number of wild and domestic birds, such as chickens, turkeys, ducks, pigeons, quails, and many other wild birds [2]. *Aspergillus fumigatus* is one of the most pathogenic fungi affecting many domestic poultry birds, where morbidity and mortality rates seem to be greater in turkeys than in chickens [3]. The genus Aspergillus is found worldwide and has a lot of different species. *Aspergillus fumigatus* and *Aspergillus niger* are two of the most common respiratory and nervous system problems [4,5].

In poultry, acute aspergillosis usually occurs in young birds, resulting in high morbidity and mortality, whereas chronic aspergillosis is more commonly observed in adult birds with less mortality [6]. Under the genus *Aspergillus*, aflatoxin-producing species such as *Aspergillus flavus*,* Aspergillus parasiticus*, and* Aspergillus nomius* are also associated with food-borne fungal infections in poultry [7]. *Aspergillus flavus *has also been previously identified in poultry feed samples from commercial poultry [8]. Aflatoxin B_1_, a mycotoxin produced by a large number of *Aspergillus* species, including *A. flavus* and *A. parasiticus*, has been described as the most potent carcinogenic mycotoxin [9]. *Aspergillus niger*, *Aspergillus nidulans, *and *Aspergillus terreus *are some other species also isolated from avian cases of aspergillosis in commercial poultry [5].

The conventional methods for identifying and detecting these fungi include cultural and morphological studies. This approach, however, is very time-consuming, laborious, and requires facilities and mycological expertise [10]. Highly variable sequences, intergenic spacers, and internal transcribed regions (ITS) from the rDNA units are widely used for molecular detection of fungal species [11]. This study used polymerase chain reaction (PCR)-based molecular detection of different types of highly pathogenic *Aspergillus* sp. by using predesigned genus-specific primers (ASAP-1 and ASAP-2) and species-specific primers (FLA-1 and FLA-2 for *A. flavus*, ASPU and Af3r for *A. fumigatus*, and ASPU and Nilr for *A. niger). *To the best of the authors’ knowledge, there is no combined report till now based on cultural and molecular studies (genus-specific and species-specific) of the abovementioned important *Aspergillus *spp. from chickens in Bangladesh. Therefore, this study was designed to isolate, molecularly detect, and determine the prevalence of the threatened *Aspergillus *spp*.* in commercial chickens.

## Materials and Methods

### Ethical statement

The experiment was approved by the Animal Welfare and Experimental Ethical Committee (AWEEC) of Bangladesh Agricultural University (BAU), Mymensingh. 

### Sample collection 

A total of 50 lung samples from diseased chickens were aseptically collected after post mortem examination from commercial farms in Kapasia Upazilla in the Gazipur district of Bangladesh. The samples were directly transported to the laboratory, maintaining a cool chain in the Department of Microbiology and Hygiene, BAU, Mymensingh.

### Culture on potato dextrose agar (PDA)

A total of 50 lung samples from *Aspergillus* affected chickens were aseptically collected after post mortem examination from commercial farms in Kapasia Upazilla in the Gazipur district of Bangladesh. The samples were directly transported to the Department of Microbiology and Hygiene Laboratory, BAU, Mymensingh, with the cool chain maintained. Inoculum prepared from lung samples was streaked onto PDA medium and incubated at 28°C for 7 days. After incubation, the colony morphology and color were recorded to identify the *Aspergillus *spp. To get pure culture, colonies of *A. flavus*, *A. fumigatus*, and *A. niger* were sub-cultured on PDA.

### DNA extraction

For the extraction for DNA from the isolated fungal sample, 500 μl lysis buffer [400 mM Tris-HCl (pH 8.0), 60 mM ethylenediaminetetraacetic acid (pH 8.0), 150 mM NaCl, 1% sodium dodecyl sulfate] was added. A small lump of mycelia from young culture was added using a sterile toothpick and kept at room temperature for 10 min.150 μl potassium acetate was added (pH 4.8; which is made of 60 ml of 5 M potassium acetate, 11.5 ml of glacial acetic acid, and 28.5 ml of distilled water). The mixture was vortexed briefly and spun down at ≥10,000 × gm for 1 min. The supernatant was transferred to a fresh Eppendorf tube and centrifuged again as described above. The supernatant was transferred into a new 1.5-ml Eppendorf tube, and an equal volume of ice-cold isopropyl alcohol was added to it. The tube was mixed by inversion briefly and stored at −20°C for 1 h. The tube was spun down at ≥10,000 × gm for 2 min, and the supernatant was discarded. The resultant DNA pellet was washed in 300 μl of 70% ethanol. The supernatant was discarded after the pellet was spun at ≥10,000 rpm for 1 min. The DNA pellet was air-dried and dissolved in 50 μl of deionized H_2_O, and 1 μl of the purified DNA was used in the PCR assay. The purified DNA was stored at −20°C for further use.

### Molecular detection by PCR

DNA was amplified for the detection of *Aspergillus *spp. using genus-specific primers ASAP-1 and ASAP-2. The *Aspergillus *spp. were then screened by PCR using species-specific primers targeting *A. flavus *using primers FLA-1 and FLA-2, *A. fumigatus *ASPU and Af3r, and *A. niger *ASPU and Nilr. The PCR products were separated by 1.5% agarose gel electrophoresis and visualized on a UV-transilluminator. The experimental results were analyzed using the Chi*-*square test by SPSS software (version 20). *p < *0.05 means a 5% level of significance, and *p < *0.01 means a 1% level of significance.

## Results

**Figure 1.**
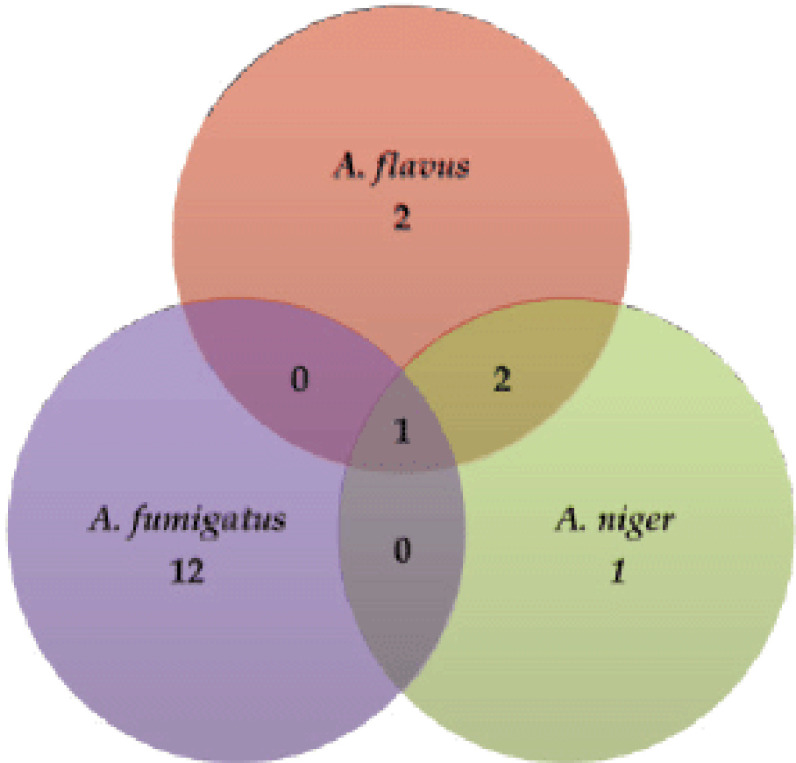
The total number of isolated fungi after cultural characterization. Samples associated with two or more fungi were also indicated.

**Figure 2.**
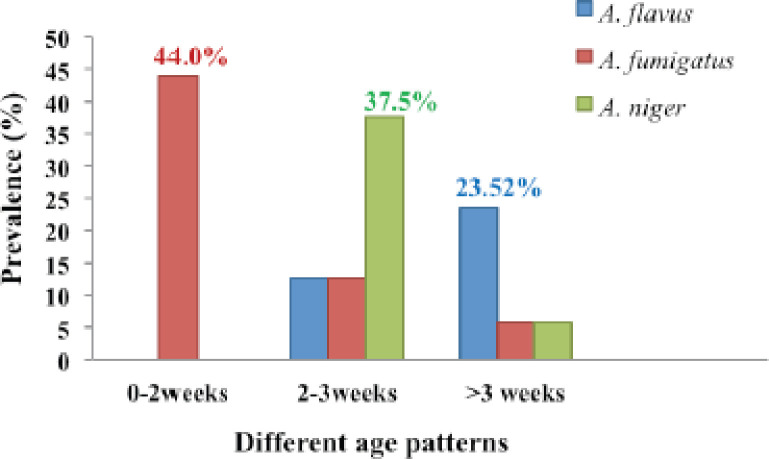
Data represents that *A. fumigatus* is mainly found at 0–2 weeks of age, whereas *A. niger *is highly found at 2–3 weeks of age, and *A. flavus *is identified at more than three weeks of age as the toxin production increases day by day.

Based on morphological studies and molecular detection by PCR, the fungus was primarily identified as *A. flavus*,* A. fumigatus*, and* A. niger*. All samples were tested, and 22 (44%) fungi were isolated (Fig. 1). *Aspergillus flavus *was detected in 10% (*n* = 5/50) of the samples. Similarly, *A. fumigatus* and *A. niger *were detected at 26% (*n* = 13/50) and 8% (*n* = 4/50), respectively, based on colony morphology and PCR assay. The prevalence *of A. flavus* in chickens more than 3 weeks of age was observed to be 23.52% (*p < *0.05). The prevalence of *A. fumigatus* causing brooder pneumonia was recorded at 44% (*p < *0.05) in chickens aged 0–2 weeks of age. *Aspergillus niger* was found to have about 37.5% at 2–3 weeks of age (*p < *0.01) (Fig. 2).

**Figure 3.**
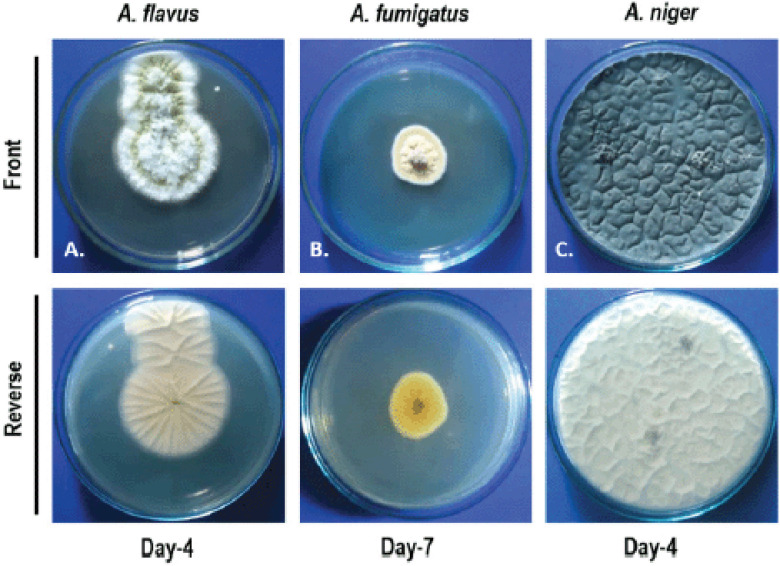
Colony characteristics of *Aspergillus *spp. (A) Powdery, olive to the lime green colony on the upper surface, and cream reverse. (B) Greenish-gray on the upper side and olivaceous gray on reverse. (C) Initially, the colony was white and gradually became black and pale yellow on the reverse. Conditions: Media: PDA Temperature: 28°C Humidity: 75%.

After a 7-day culture, colonies on PDA at 30°C were olive to lime green with a cream reverse for *A. flavus*.* Aspergillus fumigatus* produces blue-green or greenish-gray, powdery, and on the reverse is greyish ash or olivaceous gray colony (Fig. 3). *Aspergillus niger *initially produced whitish colonies, later became black, and the reverse was pale yellow.

**Figure 4.**
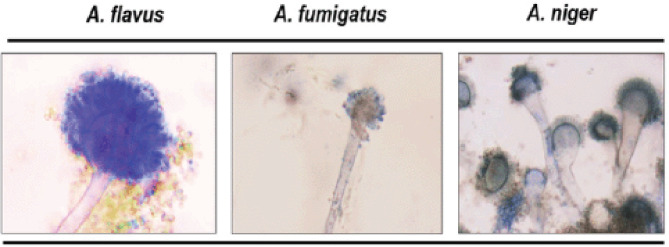
Morphology of *Aspergillus spp*. (100×).

Microscopic morphology of *Aspergillus* spp. was viewed in (100×) where *A. flavus* found as conidiophores were hyaline and coarsely roughened. Conidia grayish-green/pale green. *Aspergillus fumigatus* showed dome-shaped vesicles and blue-green heads. Conidiophores were short, smooth-walled, and had conical-shaped terminal vesicles. *Aspergillus niger* showed large, globose, dark brown conidial heads. Conidiophores were smooth-walled, hyaline, or darkened toward the vesicle (Fig. 4).

**Figure 5.**
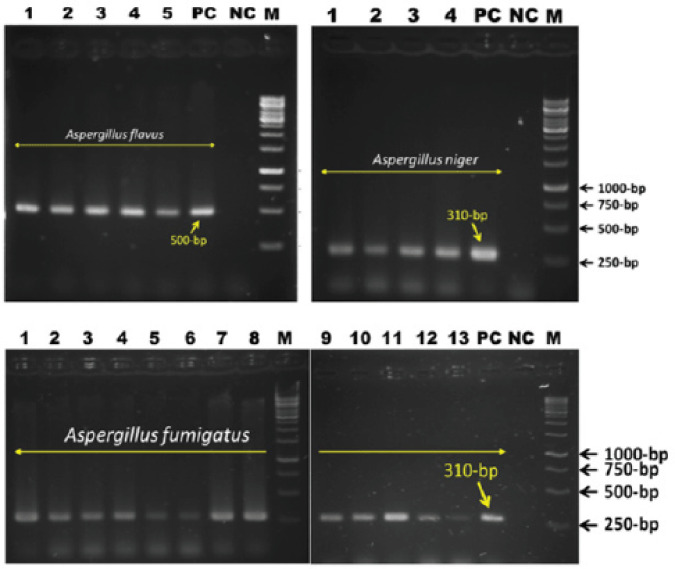
PCR assay of *Aspergillus *spp. Prevalence of *A. flavus* (10%), *A. fumigatus* (26%), and *A. niger *(8%). Genus-specific primers were used to determine all of those strains.

The PCR assay showed the different base pairs [500 base-pair (bp), 310 bp] by using species-specific primers (FLA 1 and FLA 2, ASPU and Af3r, ASPU and Nilr) for three* Aspergilli* spp., respectively (Fig. 5). Based on molecular characterization, the overall prevalence rate of *Aspergillus *spp. infection in chickens was recorded as 44%. Among the isolated fungi, *A. fumigatus* (26%) was an important cause of fungal respiratory infection in chickens, followed by *A. flavus* (10%) and *A. niger* (8%).

## Discussion

### Cultural characteristics on PDA media 

Lung samples were cultured in PDA and, after 7 days of incubation, *A. flavus *produced a powdery green (olive to lime or yellow-greyish green) with cream reverse colonies [8,12,13]. Greenish grey color colonies of *A. fumigatus *produced in PDA [14] and *A. niger* showed initial growth of whitish color colonies, which became black gradually.* Aspergillus niger *also produced a dark black colony in PDA [12,15]*.*

### Morphology study under light microscopy

Conidiophores of *A. flavus* are hyaline and coarsely roughened, with grayish-green conidia. Conidiophores were short, smooth-walled, and had conical-shaped terminal vesicles in the case of *A. fumigatus*, with a dome-shaped vesicle and blue-green heads. *Aspergillus niger* showed large, globose, dark brown conidial heads, and conidiophores were smooth-walled, hyaline, or turned dark toward the vesicle after lactophenol cotton blue staining [16,17]. 

### Molecular detection and prevalence of isolated Aspergillus

In this study, molecular detection of *Aspergillus *spp. was carried out by PCR using genus-specific primers ASAP-1 and ASAP-2 that amplified a fragment of 521 bp in length [18,19]. Specific *Aspergillus* species were identified using species-specific primers (FLA-1 and FLA-2 for *A. flavus*, ASPU and Af3r for *A. fumigatus, *and ASPU and Nilr for *A. niger*) [19–21]. Based on molecular characterization, the overall prevalence rate of *Aspergillus *spp. was 44% at the farm level, which indicates the threatening status of the poultry industry in Bangladesh. *Aspergillus* spp. was about 44.7% and 24% isolated from a chicken farm by molecular detection using ITS-1 and ITS-4 primers [1,22].

The variation of prevalence may differ due to several factors, such as the crowdiness of birds or environmental conditions. In addition, farmers may enhance the subsequent production of aflatoxins by mixing water with dry feed or dry grain. Among the isolated fungi, the prevalence of *A.* *fumigatus*,* A. flavus*, and* A. niger* was 26%, 10%, and 8%, respectively, which indicates that *A. fumigatus *was the most important cause of fungal respiratory infection in chickens. *Aspergillus fumigatus *was the most isolated one at 21.7%, followed by *A. flavus *(19.4%) and *A. niger *(17.1%) from chicken lung [1]. However, sometimes it means about 58.8% in *A.* *fumigatus *and 41.2% in *A. flavus* [23]. According to the prevalence data presented above, *A. fumigatus* is the most common species of fungal infection in poultry farms. The age of chickens is an important factor in fungal infection. In this study, we found the prevalence *of A. fumigatus*,* A. niger*, and* A. flavus *at 44%, 37.5%, and 23.52%, respectively, in different age groups of chickens (*A. fumigatus* at 0–2 weeks,* A. niger* at 2–3 weeks, and *A. flavus *at more than 3 weeks of age). The mortality rate in growing chickens was about 0.98% (> 3 to 8 weeks old) caused by aspergillosis [24], whereas the incidence was higher in chicks at about 8.27% within 1 week of age [12]. A higher morbidity rate (76%) and mortality rate (62.5%) were reported in broiler chicks at 0–2 weeks of age caused by aspergillosis [25]. 

*Aspergillus niger* may affect birds adversely with other *Aspergillus* spp., such as *A. terreus*,* A. glaucus*, and *A. nidulans *[26]. Some strains of *A. niger* have been found to produce potent mycotoxins called ochratoxins [27]. This study found that compared with other perspectives, the incidence of pathogenic and toxin-producing *Aspergilli* is a significant concern for the poultry industry in Bangladesh. This study was performed for the molecular detection of *Aspergillus* only in a specific farm area with a suspected chicken lung sample, representing a limited prevalence rate of Aspergillus infection in Bangladesh. Along with this newly adapted species-specific molecular detection, serological, and pathological experiment findings might be more confirmatory diagnostic tools to screen for *Aspergillus* infection in the poultry industry in Bangladesh. 

## Conclusion

For the first time in Bangladesh,* A. flavus*, *A. fumigatus*, and *A. niger *were successfully isolated and identified from the collected lung samples of chickens by cultural and molecular techniques. The PCR-based protocol reported in this work is a rapid and powerful tool to detect *Aspergillus* sp. Overall, the prevalence of *A. flavus*,* A. fumigatus*, and *A. niger* in chickens is obviously of great concern. Therefore, the government should take steps to maintain strict hygienic measurements and proper use of antifungals with antibiotics. Further study needs to be implemented for a more specific distribution of these species in the poultry industry in Bangladesh.

## List of abbreviations

>, Greater than; <, Less than; %, Percentage; °C, Degree celsius; BAU, Bangladesh Agricultural University; bp, Base-pair; PCR, Polymerase chain reaction.
